# Weight status and perception of body image in children: the effect of maternal immigrant status

**DOI:** 10.1186/1475-2891-11-85

**Published:** 2012-10-15

**Authors:** Emanuela Gualdi-Russo, Vanessa Samantha Manzon, Sabrina Masotti, Stefania Toselli, Augusta Albertini, Francesca Celenza, Luciana Zaccagni

**Affiliations:** 1 Department of Biomedical Sciences and Surgical Specialties, University of Ferrara, Corso Ercole I D’Este, 32, Ferrara, 44121, Italy; 2 Department of Evolutionary Experimental Biology, University of Bologna, Bologna, Italy; 3 Public Health Service Bologna, Bologna, Italy

**Keywords:** Weight status, Body image perception, Thinness, Overweight, Immigrant status

## Abstract

**Background:**

Recent studies have shown that body image perception is an important factor in weight control and may be influenced by culture and ethnicity. The aim of the present study was to assess the relationship between immigrant status of the mother and weight status and body image perception of the child.

**Methods:**

In total, 2706 schoolchildren (1405 boys and 1301 girls) aged 8–9 years and their mothers participated in a cross-sectional survey in Emilia-Romagna region (northern Italy). Weight and height of the children were measured and Body Mass Index (BMI) was calculated. Actual and ideal body image perception by the children and by the mothers with respect to their children was evaluated according to Collins’ body image silhouettes.

**Results:**

The BMI values were significantly lower in children of immigrants than in children of Italian mothers (F:17.27 vs 17.99 kg/m^2^; M:17.77 vs 18.13 kg/m^2^). The prevalence of overweight/obesity was lower, and the prevalence of underweight higher, in children of immigrant mothers than in those of Italian mothers (overweight- F:21.3 vs 29.1%; M. 28.3 vs 31.4%; underweight- F:5.16 vs 3.84%; M:6.63 vs 2.82%). The children's body image perception was consistent with the differing pattern of nutritional status. In the comparison between actual and ideal figures, the Feel-Ideal Difference Index (FID) scores resulted different between the subsample with foreign-born mother in comparison to the native one (significantly lower in daughters of immigrants) (FID- F: 0.31 vs 0.57; M: 0.35 vs 0.32). There were significant differences in the choice of the ideal figure of the child between immigrant mothers and Italian mothers (FID- F: -0.05 vs 0.19; M: -0.35 vs −0.03): the ideal figure values were higher in the immigrant mothers of male children and lower in the Italian mothers of female children.

**Conclusion:**

Our results suggest that cultural and behavioral factors linked to ethnicity play an important role in the nutritional status of children and in the perceived and ideal body image.

## Background

The prevalence of childhood overweight and obesity has increased dramatically in Western countries in the last few decades, reaching epidemic proportions and now representing a serious public health concern because of the well-known association of childhood obesity with chronic diseases such as diabetes mellitus, hypertension, cardiovascular problems and hyperlipidemia [1-6]. Furthermore, it has been suggested that there is a correlation between high BMI in childhood and permanence of obesity in adulthood, poor self-image, body dissatisfaction, social isolation, self-aggression, suicide, weight and shape concern, and development of eating disorders (anorexia nervosa, bulimia, binge eating, etc.) [6,7]. Although genetics can play an important role, environmental, cultural and social factors are the most important determinants of childhood BMI. The current literature on eating disorders and weight-related problems suggests that weight status, eating behaviors and weight concerns of children are often shaped by their parents [6-9]. The household environment influences how food and fat will be experienced. Family mealtime is often the one time of day that families spend together; therefore, it is an important moment for parents to transmit their ideas, especially those concerning food, dieting, and fatness, to their children. The most important parent in determining a child’s nutritional status, from both a genetic and environmental point of view, seems to be the mother. Maternal behavior and nutrition during pregnancy may program appetite and energy expenditure of the child, permanently affecting his hormonal, neuronal and autocrine mechanism of maintenance of energy balance [5,6]. Moreover, the mother usually makes food consumption choices for meals eaten at home, spends more time with the children and has an important role in the children’s education; it is not surprising that the mother’s attitudes to foods, weight and shape can affect the nutritional status of her children.

Recent studies have shown a relationship between childhood obesity and the immigrant status of the mother [6,9,10]. Culture and ethnicity can influence food choice and consumption. Moreover, the socio-economic status of the family can have an effect on food choice and on the possibility of engaging in physical activity and healthy behaviors [2,5,11]. Cultural ideals can also influence the relationship between BMI and body dissatisfaction among ethnic and gender groups: standards of beauty can differ among different cultures and populations, affecting the desire to be more or less thin [2,7,11-13]. Several studies have shown, for example, that Caucasian women experience body dissatisfaction at lower BMI levels than black women, whereas black women perceive themselves as normal weight when they are actually overweight [2,7]. These ethnic differences in body satisfaction and weight-related concerns can contribute differently to a malnutrition risk in native-born children and in first-generation immigrant children or ethnic minority children. Obesity and underweight are opposite extremes of the spectrum of adiposity (the latter is also responsible for a negative effect on fat free mass); both are generally quantified in terms of weight and height relative to the child’s age (BMI). Malnutrition (obesity and underweight) in childhood and adolescence is also a serious public health problem, and anorexia nervosa is one of the most common chronic conditions of adolescence in developed countries [14]. While body image perception in overweight children has been analyzed [4,15] there is a dearth of research on body image perception in underweight children.

The aim of the present study was to compare Italian and first-generation immigrant children and their respective mothers in order to analyze: i. differences in anthropometric characteristics, actual and ideal body images, and malnutrition prevalence in children with Italian or immigrant mothers; ii. the children’s body image perception and dissatisfaction versus their BMI, with particular regard to the extreme BMI categories (underweight and overweight); iii. the mother’s perception of her child’s body image and possible dissatisfaction.

In addition to the evaluation of possible ethnic trends in the subsamples, we propose a new methodology to assess the appropriateness of the body image perception in underweight children.

## Methods

A cross-sectional design was chosen and data were collected and analyzed in a sample of 2706 children aged 8–9 years attending primary school in the Emilia-Romagna region (northern Italy) during the 2004–2005 school year. The sampling methodology has been described in detail in a previous publication [16]. The sample included 1405 males and 1301 females: 12% of the children (166 males and 155 females) had an immigrant mother and the remaining 88% had an Italian mother.

Only children who received parental written consent and agreed to participate (89% of the initial sample) were allowed to take part in the study. The survey was part of a larger research project (SoNIA – Sorveglianza Nutrizionale Infanzia e Adolescenza) on the nutritional status of children and adolescent. The study was approved by the Italian Ministry of Health and was scientifically supported by the National Institute of Nutrition.

Anthropometric measurements were taken at school by trained technicians according to standardized procedures [17,18]. Height was recorded to the nearest 0.1 cm with a stadiometer and weight was measured to the nearest 0.1 kg with a high-precision mechanical scale. BMI was calculated as weight (kg) per height^2^ (m^2^). Children were classified as underweight, normal weight, overweight or obese based on international age- and sex-specific cut-off points [14,19], as reported in Table 1.

**Table 1:** **Cutoff points for body mass index for underweight**, **overweight and obesity by sex and age**[14,19]**applied to sample**

	**Underweight**		**Overweight**		**Obesity**	
**Age** (**yrs**)	**males**	**females**	**males**	**females**	**males**	**females**
8.0	14.15	14.02	18.44	18.35	21.60	21.57
8.5	14.24	14.14	18.76	18.69	22.17	22.18
9.0	14.35	14.28	19.10	19.07	22.77	22.81
9.5	14.49	14.43	19.46	19.45	23.39	23.46

Body image perception was assessed using a body silhouette chart designed by Collins [20] for preadolescent children, after eliminating facial features from silhouettes as they may influence the choice. The children were shown seven male or female silhouettes (marked F.1, F.2, F.3, F.4, F.5, F.6 and F.7), ordered in morphology from emaciation to obesity. Subjects were asked to select the silhouette which they believed was most similar to their own (‘actual’ figure) as well as the silhouette which they most desired (‘ideal’ figure). The discrepancy between the actual figure and the ideal figure represents the degree of body image dissatisfaction (FID or Feel minus Ideal Discrepancy) [21]. The FID index was computed by subtracting the score of the figure selected by children as the ideal figure from the one selected as their actual figure. A positive FID score indicates the actual figure was bigger than the ideal figure and a negative score indicates the actual figure was thinner than the ideal figure. A FID score of 0 indicates no discrepancy (same figure chosen as actual and as ideal).

The same body silhouette charts were presented to the mothers: they were asked to select the figure best representing their child and the figure they would like their child to resemble. The discrepancy between the daughter’s/son’s actual figure and the ideal figure represents the degree of the mother’s dissatisfaction (in this study indicated as mother’s FID).

**Figure 1:**
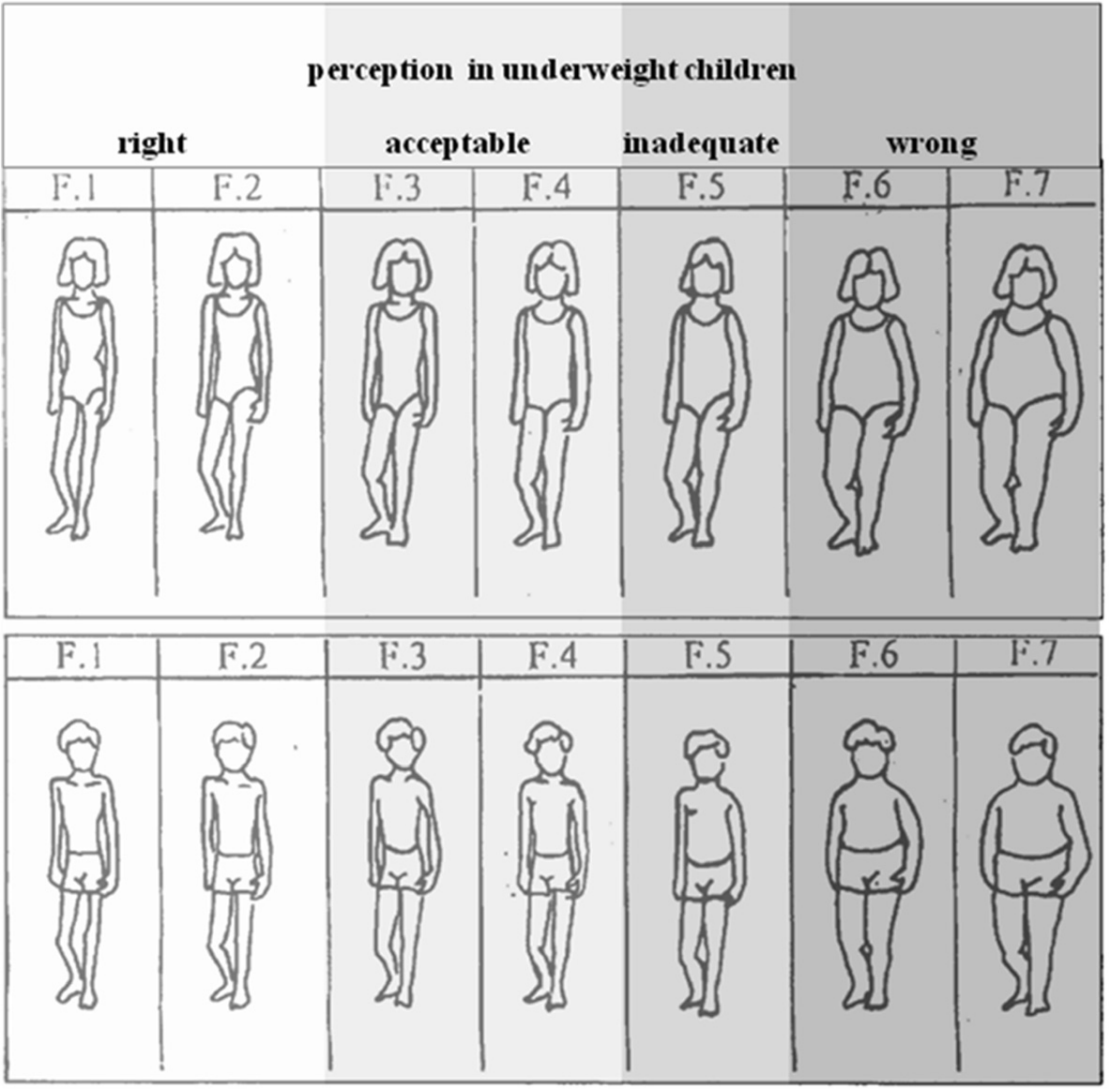
**Evaluation of body image perception in underweight children using a Body Silhouette Chart** (**reproduced with kind permission of John Wiley** &**Sons**, **Inc**.) **derived by Collins**[18]**according to the scheme**: **F**.**1**/ **correct**; **F**.**2**/ **correct**; **F**.**3**/ **acceptable**; **F**.**4**/ **acceptable**; **F**.**5**/ **inadequate**; **F**.**6**/ **wrong**; **F**.**7**/ **wrong.**

The frequencies of improper perception of body image were calculated in overweight and obese subjects and in their mothers if they chose F.1 or F.2 or F.3 as the actual figure according to the scheme proposed by Gualdi-Russo et al. [4]. In present study we propose a symmetric scheme to calculate the frequencies of improper perception in underweight subjects and in their mothers. After analysis of the self figure responses by underweight subjects, the perception of body image was so considered as (Figure 1): F.1/ correct; F.2 / correct; F.3/ acceptable; F.4/ acceptable; F.5/ inadequate; F.6/ wrong; F.7/ wrong. A range of uncertainty was allowed because of the objective difficulty a child has in figure interpretation; thus selections two places away from the correct figure were also considered acceptable *(*F.3 and F.4).

The data analysis was performed using Statistica for Windows, version 7.1 (StatSoft Italia srl, Vigonza, Padua, Italy). Data are expressed as means and standard deviations. Comparisons between two groups were performed using Student’s t-test for traits with normal distribution or the non-parametric Mann–Whitney U test. Comparisons between frequencies were performed using chi-square. Spearman’s rank-order correlations were used for ordinal data. The level of *p* < 0.05 was considered significant.

## Results

The mean weight, height and BMI values by sex and maternal birth place (Italian vs immigrant status) for the children’s sample are presented in Table 2.

**Table 2:** Anthropometric characteristics of children by sex and maternal origin

**Children**	**Italian mother**	**Immigrant mother**	**p value**
	**Mean(SD)**	**Mean(SD)**	
**Sons**	N=1239	N=166	
Weight (kg)	32.6 (7.2)	31.9 (7.8)	
Height (cm)	133.6 (6.1)	133.4 (6.9)	
BMI (kg/m^2^)	18.13 (3.11)	17.77 (3.20)	
**Daughters**	N=1146	N=155	
Weight (kg)	31.9 (7.3)	31.0 (7.0)	
Height (cm)	132.6 (6.6)	133.5 (6.2)	
BMI (kg/m^2^)	17.99 (3.15)	17.27 (2.86)	0.004

Mean weight values were slightly higher in children of Italian mothers than in those of immigrant mothers. The mean BMI value was significantly higher in daughters of Italian mothers than in those of immigrant mothers (p=0.004), as the mean weight was higher (+ 0.9 kg) and the mean height was lower (− 0.9 cm).

In both sexes (Table 3) the prevalence of overweight and obesity was higher and the prevalence of underweight was lower in children of Italian mothers than in those of immigrant mothers (overweight + obesity: M 31.4% vs 28.3%; F 29.1% vs 21.3%; underweight: M 2.82% vs 6.63%; F 3.84% vs 5.16%). In particular, sons of Italian mothers showed lower frequencies of underweight (less than half) than those of immigrant mothers. A similar tendency in underweight category was seen also in daughters. The incidence of obesity in Italian daughters was almost double than that of daughters of immigrant mothers.

**Table 3:** Weight status of children by sex and maternal origin

**BMI category**	**Italian mother**	**Immigrant mother**	**χ**^**2**^	**p value**
	**N (%)**	**N (%)**		
**Sons**	N=1239	N=166	10.75	<0.05
Underweight	35 (2.82)	11 (6.63)		
Normal weight	815 (65.78)	108 (65.06)		
Overweight	274 (22.11)	28 (16.87)		
Obese	115 (9.28)	19 (11.45)		
**Daughters**	N=1146	N=155	5.84	
Underweight	44 (3.84)	8 (5.16)		
Normal weight	768 (67.02)	114 (73.55)		
Overweight	232 (20.24)	26 (16.77)		
Obese	102 (8.90)	7 (4.52)		

The sons of Italian mothers and those of immigrant mothers seemed more likely to select F.4 and F.5, respectively, to represent their body image, even though the former had a higher BMI than the latter (Table 4). The daughters of Italian mothers and those of immigrant mothers were more likely to select F.3 and F.2, respectively, consistently with the higher values of BMI in the Italians (Table 5).

**Table 4:** Percentages of actual and ideal figures selected by males and mean BMI of children choosing each figure

**Sons**	**Italian mother**			**Immigrant mother**		
**Silhouette**	**Actual**	**Ideal**	**BMIª**	**Actual**	**Ideal**	**BMIª**
**number**	**(%)**	**(%)**	**Mean** (**SD**)	**(%)**	**(%)**	**Mean** (**SD**)
F.1	7.0	12.4	16.10 (2.07)	9.0	14.7	16.44 (2.89)
F.2	18.4	18.5	16.43 (2.07)	11.0	15.3	15.31 (1.56)
F.3	22.3	26.1	17.11 (2.03)	18.1	19.3	17.02 (1.88)
F.4	26.8	25.9	18.10 (2.35)	25.8	24.7	17.12 (2.52)
F.5	22.0	15.8	20.20 (3.17)	30.3	22.7	19.40 (3.38)
F.6	3.2	1.0	24.77 (3.63)	5.8	2.0	22.73 (3.39)
F.7	0.3	0.3	22.17 (3.19)	0.0	1.3	-

ªBMI values are referred to subjects who have chosen the figure as actual.

**Table 5:** Percentages of actual and ideal figures selected by females and mean BMI of children choosing each figure

**Daughters**	**Italian mother**			**Immigrant mother**		
**Silhouette**	**Actual**	**Ideal**	**BMIª**	**Actual**	**Ideal**	**BMIª**
**number**	**(%)**	**(%)**	**Mean(SD)**	**(%)**	**(%)**	**Mean(SD)**
F.1	9.0	17.1	15.39 (1.68)	5.4	15.4	15.59 (0.94)
F.2	24.1	32.8	16.02 (1.56)	32.9	34.3	15.75 (1.83)
F.3	27.7	28.4	17.38 (2.05)	26.8	23.8	16.68 (1.63)
F.4	22.7	17.6	18.94 (2.48)	24.8	23.1	18.55 (3.06)
F.5	14.5	3.6	21.54 (3.17)	8.1	2.8	21.23 (2.52)
F.6	1.9	0.5	24.93 (2.76)	2.0	0.7	24.50 (2.24)
F.7	0.1	0	22.55	0	0	-

ªBMI values are referred to subjects who have chosen the figure as actual.

The children’s body image perception was consistent with the differing pattern of nutritional status. Tables 4 and 5 show an increase in mean BMI value with increasing value of the silhouette; self figure perception and BMI were significantly correlated (p<0.001) in both sexes (r =0.53 for sons of Italian mothers; r =0.47 for sons of immigrant mothers; r =0.66 for daughters of Italian mothers; r =0.55 for daughters of immigrant mothers).

These results provide a proxy measure of general appropriateness of the children’s body image perception, even though there was a tendency (as previously pointed out by comparing average scores of body image in the two male subsamples) to the selection of thinner figures (with respect to their BMI) in children of Italian mothers or of thicker figures in sons of immigrant mothers.

The higher BMI variability (SD) in children (especially in males) who selected F.6 as the actual figure was probably due to a tendency by most obese children to avoid the last fat figure (F.7). As a result, only 0.2% of the children selected F.7.

In the comparison between the actual and ideal figure mean scores (Tables 6, 7), the children of both sexes showed a preference for thinner bodies, with lower mean values of the ideal figures than the actual figures. However the FID values only differed significantly between the daughters of Italian mothers and those of immigrant mothers, being higher in the former group.

**Table 6:** Body Silhouette Chart responses in sons by maternal origin

**Sons**	**Italian mother**	**Immigrant mother**	**p value**
	**Mean(SD)**	**Mean(SD)**	
Actual figure	3.49 (1.31)	3.75 (1.38)	0.0139
Ideal figure	3.18 (1.29)	3.37 (1.47)	
Son’s FID	0.32 (1.41)	0.35 (1.61)	
Mother’s actual	3.44 (1.29)	3.46 (1.33)	
Mother’s ideal	3.47 (0.94)	3.72 (1.02)	0.0049
Mother’s FID	−0.03 (0.94)	−0.35 (1.37)	0.0059

**Table 7:** Body Silhouette Chart responses in daughters by maternal origin

**Daughters**	**Italian mother**	**Immigrant mother**	**p value**
	**Mean(SD)**	**Mean(SD)**	
Actual figure	3.16 (1.25)	3.03 (1.14)	
Ideal figure	2.59 (1.10)	2.66 (1.12)	
Daughter’s FID	0.57 (1.21)	0.31 (1.37)	0.0181
Mother’s actual	2.96 (1.30)	2.90 (1.35)	
Mother’s ideal	2.83 (0.92)	2.96 (0.92)	
Mother’s FID	0.19 (0.89)	−0.05 (1.29)	0.0363

The mother’s perception of her child’s appearance usually corroborated her child’s body perception in both samples, with slight and non significant differences between the mean responses of parent and child. However the Italian mother’s desire (Table 7) that her daughter be thin was not as strong as the daughter’s desire, while the mother’s perceived figure of her son was the same as her choice of his ideal figure, as shown by the low FID values. Instead the immigrant mother’s perceived figure of her daughter was the same as her choice of her ideal figure, whereas she desired that her son be “fatter”, as shown by the FID value. Therefore, the main effect of ethnicity was significant for the FID index in the mothers subsamples, showing a greater dissatisfaction in Italian mothers with their daughters and in immigrant mothers with their sons (p<0.05).

**Figure 2:**
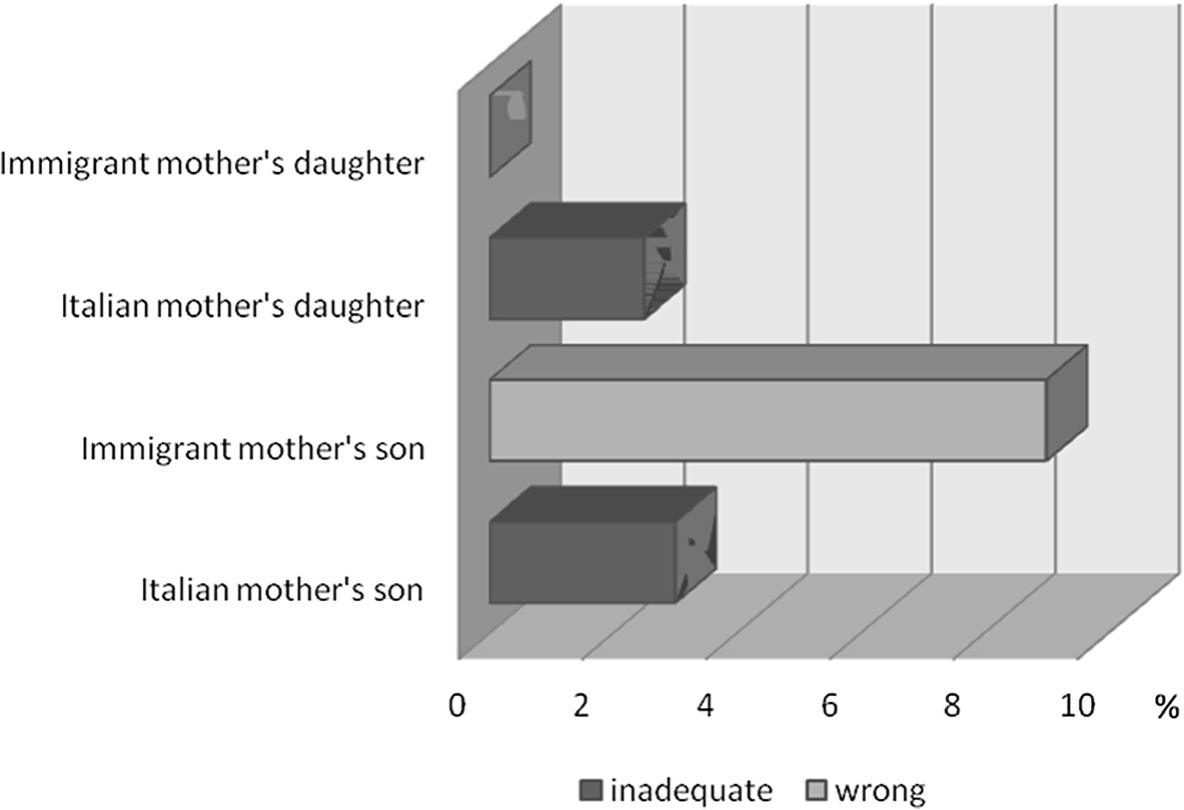
Frequency of inadequate or wrong body image perception in underweight children by sex and maternal origin.

**Figure 3:**
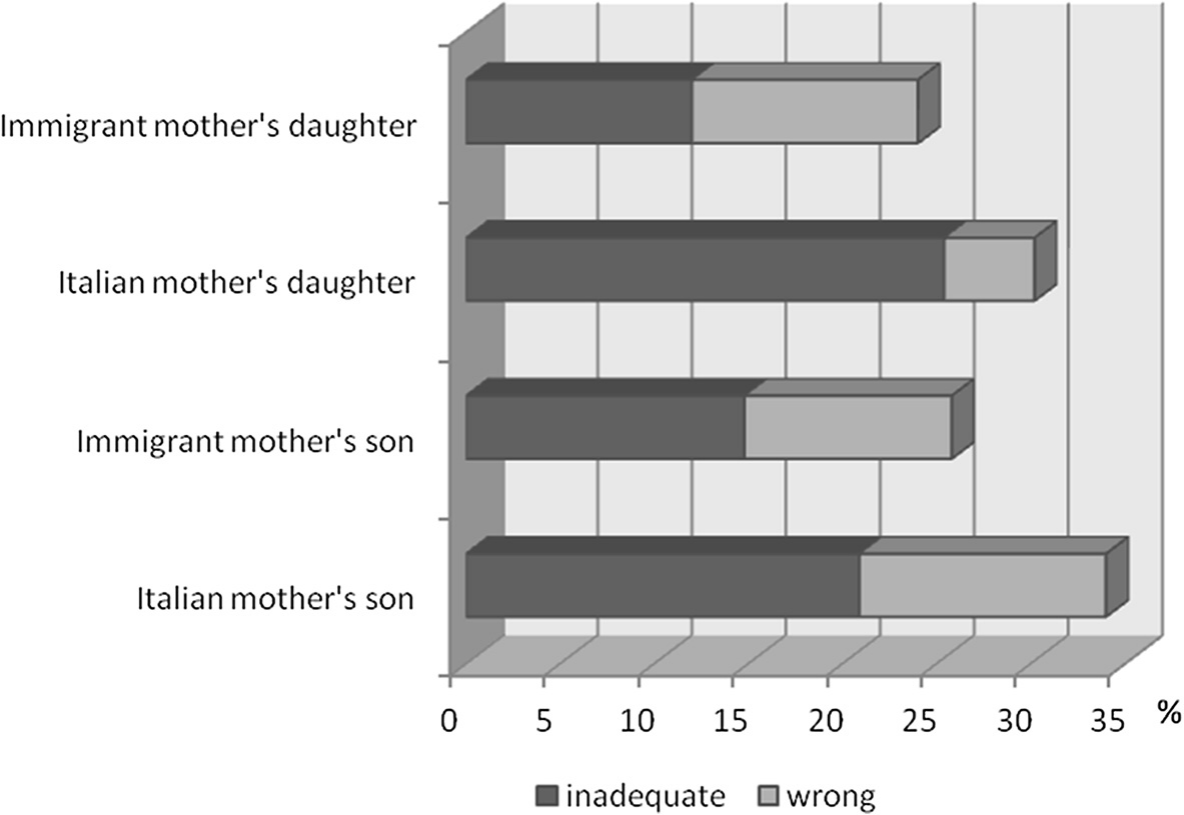
**Frequency of inadequate or wrong body image perception in overweight**/**obese children by sex and maternal origin.**

When the malnourished children were analyzed according to the new scheme proposed for underweight (Figure 1) or the scheme previously proposed for overweight [4], it was found that the frequencies of inadequate or wrong body image perception in underweight sons of Italian mothers were one third than in sons of immigrant mothers (M: 3.03% vs 9.09%), while the opposite occurred in daughters (F: 2.3% vs 0%) (Figure 2). The frequencies of inadequate or wrong body image perception in overweight/obese children of Italian mothers were higher than those in children of immigrant mothers (M: 34% vs 25.9%; F: 30.2% vs 24%) (Figure 3). Among mothers of overweight/obese children with inadequate or wrong body image perception, 50% of Italian mothers and 67% of immigrant mothers had an incorrect perception of their daughters, while only 16% of Italian mothers and 29% of immigrant mothers showed an incorrect perception of their sons. No maternal misperception was found among mothers of underweight children with inadequate or wrong body image perception.

## Discussion

In this study we sought to detect and evaluate eventual differences in weight status and in body image perception in schoolchildren living in Emilia Romagna region (North Italy), and in their mothers perception of them, in relation to the immigrant or Italian status of the mother. In addition we sought to develop and explore a new scheme to validate the body image perception in underweight children.

Previous studies have reported a relationship between the nutritional status of children and the immigrant and nutritional status of their mothers [5,6,9,22-24]. In addition to genetic influences or events occurring during fetal life, great importance is given to the mother's behavior. Indeed the mother's behavior concerning food serves as a model for her children and strongly influences their feeding behavior. Moreover, the mother usually deals with the selection and preparation of food for meals eaten at home and thus is responsible for the accessibility or not to certain foods by her children, for eventual food restrictions or for any pressure to eat certain foods because they are considered healthy or to eat more or less in general [8,10,23-25].

Differences in the choice and preparation of food, and in eating habit in general, are often linked to ethnic and cultural traditions: these differences will probably have an impact on the diet and nutritional status of children.

Our findings support a greater prevalence of overweight and obesity in children with Italian mothers and a higher prevalence of underweight in children with immigrant mothers than in their peers. This may be related to genetic factors, but also perhaps to a lower socio-economic status of families recently immigrated to Italy, with preservation of the culinary traditions of the country of origin and consequently with lower accessibility by children of immigrants to certain types of foods rich in fat.

The analysis of body image perception showed that the children’s choice of the actual silhouette tended to be consistent with their nutritional status, as assessed by the anthropometric survey (BMI). While these data do not prove a correct body image perception by the children, they provide a proxy measure of its general appropriateness, as it approaches their actual nutritional status.

On average the children, both with Italian and immigrant mothers and both males and females, chose an “ideal” silhouette that was slimmer than the one chosen as “actual”: this means that they would like to be slimmer than they perceive themselves, with a consequent positive FID. This was particularly evident for daughters of Italian mothers, who showed a significantly higher FID than daughters of immigrant mothers, in agreement with the findings of Thompson et al. [1] and Caradas et al. [25].

The daughters of Italian mothers were generally the most dissatisfied (their FID was almost twice that of all the other subsamples), especially the overweight and obese daughters who chose mean actual figure values of 4.09 and 4.82, respectively, but would have liked to be (ideal figure) 2.81 and 2.93 on average. These data confirm previous studies in which Italian girls were more dissatisfied than Italian boys [4] and confirm general data according to which immigrant women (particularly black ones) are more satisfied with their body image than white ones [2,26]. Most of the males who would like to be slimmer were the obese sons of Italian mothers.

Analysis of the body image perception the mothers had of their children showed that they had a tendency to perceive them as thinner than the children perceived themselves. Moreover the mothers were more satisfied with their children’s body image than the children were, especially the immigrant mothers of girls and the Italian mothers of boys. Instead, the Italian mothers of girls were the only ones to have a positive FID, meaning they would like their daughters to be slimmer than they perceive them. However, the positive FID of Italian mothers was lower than the FID of their daughters, indicating that the mothers were more lenient about the nutritional status of their daughters than the daughters themselves.

In contrast, the immigrant mothers of boys had a negative FID, meaning they would like their sons to be heavier than they perceive them: immigrant mothers chose F.4 as the ideal figure for their sons, whereas Italian mothers preferred F.3. These data disagreed with the FID values of their children, which were positive to the same degree as those of the mothers were negative (sons’ FID= + 0,35; mothers’ FID= −0,35).

The fact that the Italian mothers significantly preferred that their daughters be slimmer whereas the immigrant mothers significantly preferred that their sons be heavier may be linked to socio-cultural and behavioral influences, perhaps related to different aesthetic ideals.

A particular analysis of body image discrepancy was carried out on malnourished children on the basis of two schemes applied to Collins’ silhouettes: the first was previously proposed for overweight and obese children [4], the second is a new scheme proposed in this study for underweight children. An interesting result of this analysis is a much higher (triple) frequency of body image discrepancy in underweight sons of immigrant mothers than in sons of Italian mothers. The opposite was observed in girls, in whom only daughters of Italian mothers showed misperception (albeit with low frequency). A very different situation was found in overweight and obese children, with frequencies of body image discrepancy greater than 30% in the children of Italian mothers and around 25% in those of immigrant mothers.

Previous studies [22,24,25,27-29] and our findings support the tendency in overweight subjects to underestimate their nutritional status. This distortion of body image perception in overweight children may be exacerbated by the perception and beauty ideals of the parents (mothers in particular). Indeed the mothers of overweight/obese children showed a high percentage of misperception, particularly with regard to daughters.

This situation is serious and can have negative effects on health, as body image misperception in overweight and obese children and in their parents may prevent the assumption of correct dietary and exercise habits. On the other hand, misperceptions and distortions of body image can also lead to opposite behaviors, for example to excessive weight control and eating disorders such as anorexia and bulimia [4,30,31]. On the other hand, the presence of overweight male individuals who see themselves as very slim, who are satisfied with their body image and choose as ideal a silhouette heavier than that corresponding to their nutritional status may be related to the erroneous belief that heavier figures correspond to greater muscularity [4,32]. These ideal body images, also seen in mothers (especially immigrant mothers) regarding their sons, are in agreement with findings by Jeffery et al. [33], according to which parents were less likely to identify overweight in sons than in daughters.

Concerns about nutritional status, weight and body image also seem to show a close relationship with ethnicity, being largely influenced by cultural models and standards of beauty that can vary among different cultures. The importance of cultural and ethnic component is made explicit by the different ideals of beauty: larger women are considered healthy and more attractive in many traditional African and Latin American cultures [2,34-36], unlike what usually occurs in Western countries. Differences in how immigrant and Italian women perceive their bodies and those of their children, and cultural patterns of beauty underlying them, may have an impact on the way they feed and control their weight and that of their children. It must not be forgotten that the mothers’ feeding behavior acts as a direct model for the feeding behavior of the children [24]. These differences in ideals of beauty may be the source of the greater dissatisfaction with the nutritional status of their daughters shown by Italian mothers than by immigrant mothers, who instead would like their sons to be heavier. Moreover, the body image dissatisfaction in children (particularly in girls) can have serious psycho-social consequences, with low self-esteem, acquisition of risk behaviors and emergence of eating disorders during adolescence (with a tendency to anticipation of the age of onset of the disorders into childhood) [21,37].

Some limitations of the present study should be noted: the lack of precise information on the country of origin of the immigrant mother and on the socio-economic status of the families. Furthermore, despite evidence of the close relationships between weight status of mother and child [8,10], we did not have the chance to collect anthropometric data for the mothers.

Despite these limitations, this study found differences in nutritional status and body image perception (and satisfaction) of two subsamples of children with Italian or immigrant mothers, and also in their mothers; these differences are probably due to socio-cultural and behavioral influences linked to ethnic origin.

It can be reasonably supposed that the ethnic heterogeneity of the sample, given its width, resembles that which can be found in literature for Emilia Romagna students [38], having a higher frequency of Moroccans (20.7%), then Albanians (15.6%), followed by Romanians, Chinese and Tunisians.

From a socio-economic point of view [39], immigrant families are generally concentrated in the lower social strata. According to Italian labor demand, primarily oriented toward unskilled workers, a large numbers of working immigrants are employed in unskilled jobs with low salaries, although some hold high educational qualifications (especially those coming from Eastern Europe and Asia) [40,41]. In addition, another element which have influenced their behaviors and habits (life style, diet etc.) may be the religious diversity, including a 49.1% of Christians, 33.2% of Muslims and different Eastern religions (about 5%) [42].

In addition to differences in diet and exercise habits between ethnic groups, our results confirm the possibility of nutritional status disparities [43] that could be related to differences in body image, leading to possible differences in responses to weight changes and weight control.

These results indicate the need to monitor and control nutrition, BMI, body image perception and body image satisfaction in children, especially those in BMI categories of underweight or overweight and obesity. Parental involvement in monitoring is crucial in order to establish strategies of weight control and to supervise the nutritional status of children, with the aim of bringing it within recommended ranges.

Use of Collins’ body silhouette chart according to the proposed methodology for assessing body perception and misperception in underweight and overweight children may be a rapid tool for the assessment and prevention of health risk behaviors, including eating disorders.

## Conclusions

The association of a quantitative approach (evaluation of anthropometric data) and of a qualitative one (evaluation of body image perception) has proved to be sensitive in the examined multi-ethnic samples. This confirms the importance of this approach in providing information on the nutritional status of the child in relation to body image perception and in identifying any hazardous health situation to be corrected and monitored.

## Competing interests

The authors declare that they have no competing interests.

## Authors’ contributions

EG-R, ST, AA and LZ were responsible for conception of the study, study design and set up. AA and FC were responsible for data collection. LZ, FC and SM entered and analyzed the data. LZ, VM and SM drafted the manuscript. All authors were involved with data interpretation, critical revisions of the paper and provided approval for its publication. All authors read and approved the final manuscript.
